# Private equity ownership and artificial intelligence adoption by hospitals in the United States

**DOI:** 10.1093/haschl/qxag261

**Published:** 2026-09-25

**Authors:** Rocco Friebel, Daniel Arnold, Michael Anderson, Jonathan Cantor, Yashaswini Singh

**Affiliations:** Department of Health Policy, The London School of Economics and Political Science, Houghton Street, London WC2A 2AE, United Kingdom; LSE Health, The London School of Economics and Political Science, Houghton Street, London WC2A 2AE, United Kingdom; Department of Health Services, Policy, and Practice, Brown University, 121 S Main Street, Providence, RI 02903, United States; Department of Health Policy and Management, Columbia University, 722 West 168th Street, New York City, NY 10032, United States; Department of Health Services, Policy, and Practice, Brown University, 121 S Main Street, Providence, RI 02903, United States; LSE Health, The London School of Economics and Political Science, Houghton Street, London WC2A 2AE, United Kingdom; Centre for Primary Care and Health Services Research, The University of Manchester, Oxford Road, Manchester M13 9PL, United Kingdom; RAND Corporation, 1778 Main Street, Santa Monica, CA 90401, United States; Department of Health Services, Policy, and Practice, Brown University, 121 S Main Street, Providence, RI 02903, United States

**Keywords:** private equity, artificial intelligence, hospital ownership, technology adoption

## Abstract

**Introduction:**

Private equity (PE) firms own a growing share of United States hospitals and claim that capital infusions support technology investment. We examined the association between PE hospital ownership and the likelihood of artificial intelligence (AI) technology adoption.

**Methods:**

We linked the 2024 American Hospital Association Annual Survey to a database of PE acquisitions and exits. Mixed-effects logistic models estimated any, clinical, and operational AI adoption by hospital ownership type, adjusting for hospital and market characteristics. In secondary analyses, we examined challenges to AI implementation.

**Results:**

PE-owned hospitals had higher adjusted adoption of any AI (difference, 24.5 percentage points; 95% confidence interval, 11.2 to 37.8) and clinical AI (difference, 27.5 percentage points; 95% confidence interval, 14.0 to 41.1) than other for-profit hospitals but did not differ from not-for-profit hospitals. Operational adoption did not differ by ownership. In secondary analyses, not-for-profit hospitals reported any, cost and resource, and regulatory and safety challenges more often than PE-owned hospitals; PE-owned hospitals reported staff-training challenges more often than other for-profit hospitals.

**Conclusion:**

PE-owned and not-for-profit hospitals had similar adoption of any and clinical AI, and both exceeded other for-profit hospitals, consistent with a capital access advantage that does not extend to the broader for-profit sector.

Key Points

- Private equity-owned and not-for-profit hospitals had similar adjusted adoption of any and clinical artificial intelligence, and both exceeded other for-profit hospitals.
- Operational adoption did not differ significantly by ownership.
- Because the analysis is cross-sectional, it cannot establish whether private equity ownership itself drives higher adoption.

## Introduction

Private equity (PE) firms have acquired more than 488 U.S. hospitals, including general acute care, psychiatric and rehabilitation, and children's hospitals.^1,2^ Emerging evidence on the impact of PE ownership on hospital performance has been mixed,^3^ including effects on staffing,^4-7^ quality of care,^8-10^ and patient experience.^11^ In addition, several studies document financial strategies, including sale-leaseback transactions of hospital real estate, that may impact long-term operational capacity.^12-14^ While proponents of PE investments highlight the potential benefits of capital infusions that can facilitate technological investments,^15-17^ the association between PE investments in health care and technological adoption remains unexamined.

Understanding PE's role in facilitating technology investments is particularly salient given recent expansion of AI models in health care.^18,19^ AI tools in health care, including clinical decision support systems and operational optimization platforms,^20-22^ can reduce administrative burden; however, early evidence indicates the propensity of AI-assisted hospital billing to increase health care spending.^23^ At the same time, AI implementation in hospitals typically requires substantial upfront capital, often $2.5-$5 million, and 18-24 months before producing a positive return on investment.^24,25^ Competing theories emerge on how PE hospitals may approach AI implementation. On the one hand, PE investments may facilitate AI adoption by providing capital to hospitals with limited financial resources and managerial expertise that may facilitate revenue optimization.^26^ On the other hand, PE investments can defer long-term, non-revenue generating investments like AI because of their shorter investment horizons and debt service pressures that may constrain discretionary spending.^27^

Hospital ownership may shape both the capacity and incentives to adopt technology. Previous research shows not-for-profit hospitals respond to financial incentives in a similar manner as for-profit counterparts,^28^ and recent studies have found not-for-profit hospitals adopted AI and health information technologies more frequently than for-profit hospitals, particularly for clinical applications.^29-31^ Conversely, for-profit hospitals may respond more strongly to service profitability.^32^ In particular, PE-acquired hospitals have selectively added profitable technologies such as robotic surgery and digital mammography.^33^ These findings motivate separating PE-owned hospitals from both other for-profit and not-for-profit hospitals.

We therefore examined the cross-sectional association between hospital ownership and AI adoption by linking PE acquisition data to the 2024 American Hospital Association (AHA) Annual Survey, released in 2026. We compared three mutually exclusive groups: PE-owned, other for-profit, and not-for-profit hospitals.

## Data and methods

### Data and sample

We identified 555 hospitals ever acquired by PE firms between 2000 and 2024 using the database constructed by Kim and colleagues from six commercial deal sources.^34^ We retained the full acquisition and exit history and defined active PE ownership in 2024 directly from the most recent acquisition or exit record. After linkage, 224 hospitals were classified as actively PE-owned under the Kim et al. deal-record definition.

We linked acquisition records to the 2024 AHA Annual Survey using standardized Centers for Medicare & Medicaid Services Certification Numbers. The 2024 survey, released in 2026, was the first AHA Annual Survey to collect self-reported information on clinical and operational AI adoption.^35^

We restricted the linked sample to general acute care hospitals with complete ownership information (*n* = 4305). The primary analytic sample included 2290 hospitals that provided a substantive response to at least one AI adoption question. Response was 31.3% among PE-owned hospitals, 25.2% among other for-profit hospitals, and 57.7% among not-for-profit hospitals (Table 1).

**Table 1.** Artificial intelligence module response and adoption by hospital ownership.

Measure	PE-owned	Other for-profit	Not-for-profit, including government	*P* value: PE vs NFP	*P* value: PE vs other FP
Linked hospitals, No.	224	417	3664		
AI-module response, No. (%)	70 (31.3)	105 (25.2)	2115 (57.7)	<0.001	0.100
*Hospital characteristics among AI-module respondents*					
Admissions, mean (SD)	7421.4 (8093.6)	9017.8 (14 638.5)	9295.6 (12 918.0)	0.066	0.356
Staffed beds, mean (SD)	151.2 (146.1)	181.3 (269.8)	203.5 (262.3)	0.006	0.343
County HHI, mean (SD)	5875.7 (3237.4)	4391.7 (3516.0)	6020.1 (3510.1)	0.715	0.005
Hospital size, n/N (%)				0.033	0.290
Small (<100 beds)	34/70 (48.6)	56/105 (53.3)	1015/2115 (48.0)		
Medium (100-300 beds)	28/70 (40.0)	31/105 (29.5)	613/2115 (29.0)		
Large (>300 beds)	8/70 (11.4)	18/105 (17.1)	487/2115 (23.0)		
Rural, *n*/*N* (%)	6/70 (8.6)	14/105 (13.3)	465/2115 (22.0)	0.007	0.332
Teaching, *n*/*N* (%)	21/70 (30.0)	30/105 (28.6)	851/2115 (40.2)	0.085	0.839
Accredited, *n*/*N* (%)	59/70 (84.3)	76/105 (72.4)	1347/2115 (63.7)	<0.001	0.066
U.S. Census region, *n*/*N* (%)				<0.001	0.447
Northeast	1/70 (1.4)	1/105 (1.0)	309/2115 (14.6)		
Midwest	5/70 (7.1)	16/105 (15.2)	781/2115 (36.9)		
South	58/70 (82.9)	80/105 (76.2)	588/2115 (27.8)		
West	6/70 (8.6)	8/105 (7.6)	432/2115 (20.4)		
Answered at least one challenge item, among AI module respondents, *n*/*N* (%)	69/70 (98.6)	100/105 (95.2)	2048/2115 (96.8)	0.723	0.404
*AI adoption among AI-module respondents*					
Any AI adoption, *n*/*N* (%)	22/70 (31.4)	9/105 (8.6)	718/2115 (34.0)	0.661	<0.001
Clinical AI adoption, *n*/*N* (%)	22/68 (32.4)	7/103 (6.8)	689/2104 (32.7)	0.946	<0.001
Operational AI adoption, *n*/*N* (%)	5/65 (7.7)	6/96 (6.3)	278/2044 (13.6)	0.169	0.758

Module response uses all 4305 linked hospitals as denominators. Hospital characteristics and adoption outcomes are reported among AI-module respondents. Comparisons use Welch t tests for continuous variables and χ^2^or Fisher exact tests for categorical variables. Hospital-size and region *P* values compare the complete distributions.

### Study variables

The exposure was a three-category ownership variable: actively PE-owned according to the Kim et al. deal record, other for-profit, or not-for-profit (including government) as of 2024. The primary outcome was full integration of AI in at least one clinical or operational domain. Clinical domains included diagnostics, predictive analytics, decision support, surgery, patient communication, population health management, and emergency resource allocation. Operational domains included revenue cycle management, supply chains, workforce management, patient flow, demand forecasting, and operational efficiency (see Supplementary Material 1).

Exploring, pilot/testing, and expanding responses were not coded as adoption, and “don’t know” responses were treated as missing rather than non-adoption in the primary analysis; hospitals with no substantive AI-item response were excluded from the primary analysis.

In secondary analyses, we examined six reported implementation challenges: cost and resource requirements, staff training and upskilling, data integration and interoperability, regulatory compliance and safety concerns, patient trust and acceptance, and “other” barriers. For each challenge, the analytical sample comprised hospitals with a nonmissing response to that item; unanswered items remained missing, and a wholly blank challenge grid was not classified as absence of challenges. Separately, using all linked hospitals, we modeled the probability of answering at least one challenge item.

### Statistical analysis

We compared AI response and unadjusted adoption across ownership groups using chi-square tests, replacing them with Fisher exact tests when expected cell counts were below five.

We estimated one mixed-effects logistic regression for each adoption outcome using the three-category ownership exposure and state random intercepts. Models adjusted for hospital size, admissions (per 1000), teaching status, accreditation, rural location, and market concentration (Herfindahl–Hirschman index). We report ownership-specific adjusted probabilities and pairwise percentage-point differences with 95% confidence intervals. Bonferroni adjustment was applied to the three ownership differences within each outcome. Implementation-challenge models were secondary and exploratory and used challenge-specific respondents.

Sensitivity analyses examined alternative nonresponse coding; strict and ordinal outcome definitions; inverse-response weighting; region fixed effects; reduced and Firth penalized-likelihood specifications; health-system-clustered inference; exclusion of formerly PE-owned hospitals, and propensity score matching (Supplementary Materials 2-6).

The propensity-score analysis compared PE-owned hospitals with other for-profit controls using 1:1, 3:1, and 5:1 nearest-neighbor matching with replacement, common support, and prespecified calipers (0.10); balance was assessed using standardized differences.^36^

## Results

Of 4305 acute care hospitals with complete ownership information, 2290 (53.2%) answered at least one AI item and were included in our sample.

Unadjusted adoption of any AI application was 31.4% in PE-owned hospitals, 8.6% in other for-profit hospitals, and 34.0% in not-for-profit hospitals. Corresponding clinical adoption rates were 32.4%, 6.8%, and 32.7%; operational adoption rates were 7.7%, 6.3%, and 13.6%, respectively (Table 1).

In adjusted analyses, the probability of any AI adoption was 33.3% (95% CI, 19.8% to 46.9%) for PE-owned hospitals, 8.8% (95% CI, 2.6% to 15.1%) for other for-profit hospitals, and 32.4% (95% CI, 25.5% to 39.3%) for not-for-profit hospitals (Table 2; Figure 1).

**Figure 1.**
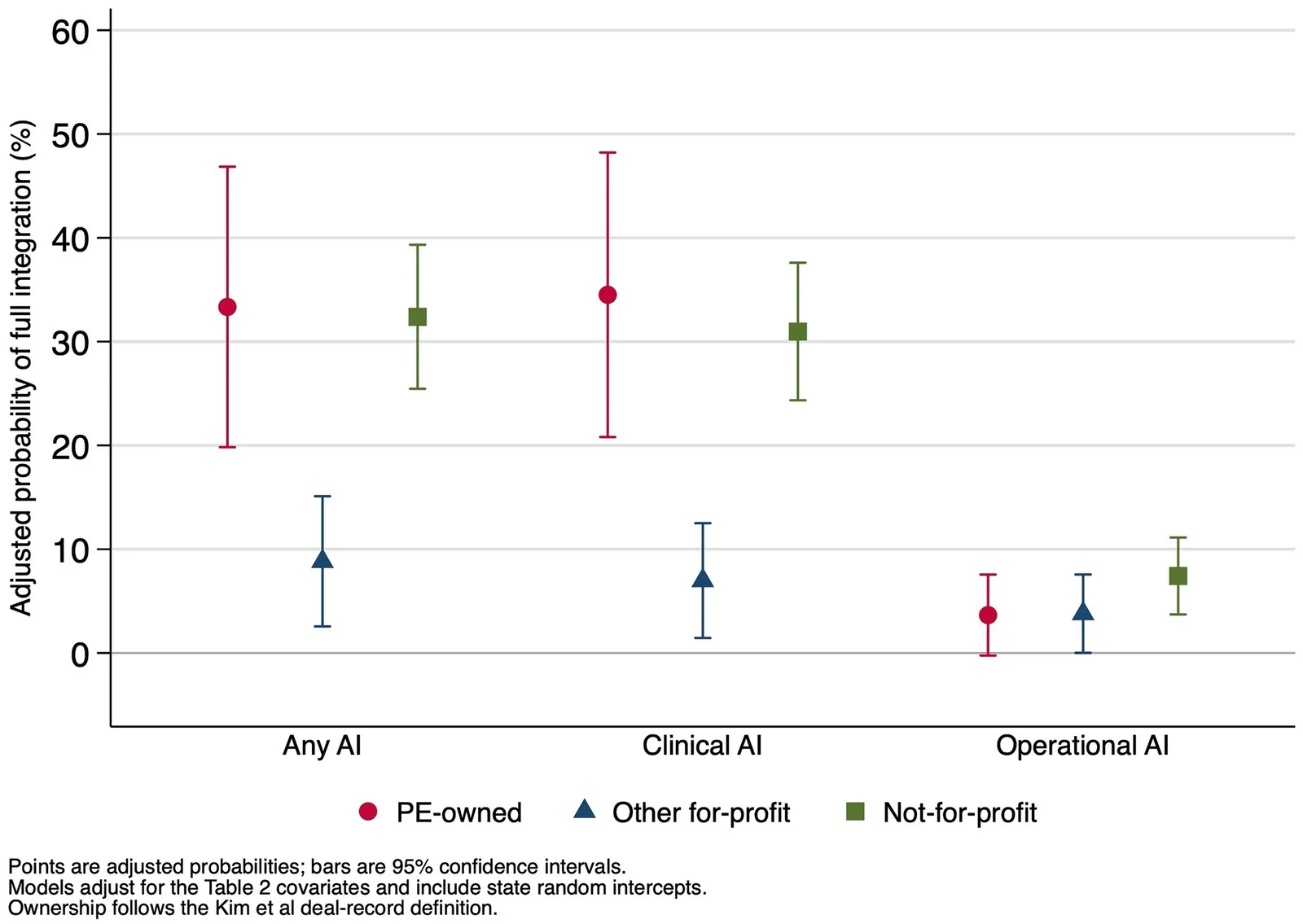
Adjusted probability of artificial intelligence adoption by hospital ownership and use domain. Probabilities derive from separate mixed-effects logistic regressions for any, clinical, and operational adoption, with state random intercepts and adjustment for hospital size, admissions, teaching status, accreditation, rural location, and market concentration. Error bars show 95% confidence intervals. PE indicates private equity.

**Table 2.** Adjusted artificial intelligence adoption by hospital ownership and use domain.

Outcome	PE-owned, % (95% CI)	Other FP, % (95% CI)	NFP, % (95% CI)	PE vs other FP, pp (95% CI); *P* value	PE vs NFP, pp (95% CI); *P* value	NFP vs other FP, pp (95% CI); *P* value
Any AI (*n* = 2290)	33.3 (19.8 to 46.9)	8.8 (2.6 to 15.1)	32.4 (25.5 to 39.3)	24.5 (11.2 to 37.8); <0.001	0.9 (−10.9 to 12.8); 0.875	23.6 (16.3 to 30.9); <0.001
Clinical AI (*n* = 2275)	34.5 (20.8 to 48.2)	7.0 (1.5 to 12.5)	31.0 (24.3 to 37.6)	27.5 (14.0 to 41.1); <0.001	3.5 (−8.6 to 15.7); 0.568	24.0 (17.0 to 31.0); <0.001
Operational AI (*n* = 2205)	3.7 (−0.2 to 7.6)	3.8 (0.0 to 7.6)	7.4 (3.7 to 11.1)	−0.1 (−4.7 to 4.4); 0.955	−3.8 (−7.7 to 0.2); 0.060	3.6 (−0.1 to 7.4); 0.058

Ownership follows the Kim et al. deal-record definition. Estimates derive from mixed-effects logistic regressions with state random intercepts, adjusted for hospital size, admissions, rural location, teaching status, accreditation, and market concentration. Confidence intervals and *P* values shown in the table are unadjusted Wald estimates. Statistical significance statements in the text use Bonferroni-adjusted *P* values for the three ownership contrasts within each outcome. Intervals extending below zero reproduce the Wald output and are not truncated. Contrasts are shown as first-listed group minus second-listed group.

AI, artificial intelligence; CI, confidence interval; FP, for-profit; NFP, not-for-profit, including government hospitals; PE, private equity; pp, percentage points.

PE-owned hospitals had a higher probability of any AI adoption than other for-profit hospitals (difference, 24.5 percentage points; 95% CI, 11.2 to 37.8; *P* < 0.001) and did not differ from not-for-profit hospitals (difference, 0.9 percentage points; 95% CI, −10.9 to 12.8; *P* = 0.875). Not-for-profit hospitals also had higher adoption than other for-profit hospitals (difference, 23.6 percentage points; 95% CI, 16.3 to 30.9; *P* < 0.001). The PE vs other for-profit difference remained significant after Bonferroni adjustment (*P* = 0.001).

For clinical AI, adjusted probabilities were 34.5% for PE-owned, 7.0% for other for-profit, and 31.0% for not-for-profit hospitals. PE-owned hospitals had higher clinical adoption than other for-profit hospitals (difference, 27.5 percentage points; 95% CI, 14.0 to 41.1; *P* < 0.001) but did not differ from not-for-profit hospitals. For operational AI, adjusted probabilities were 3.7%, 3.8%, and 7.4%, respectively, with no statistically significant pairwise differences.

Unadjusted challenge responses across 4305 linked hospitals differed by ownership: 35.7% of PE-owned, 25.4% of other for-profit, and 58.1% of not-for-profit hospitals answered at least one challenge item, and adjusted probabilities were 35.3%, 22.9%, and 58.5%, respectively (Table 3; Figure 2). After Bonferroni adjustment, not-for-profit hospitals were more likely than PE-owned hospitals to report any challenge, cost and resource challenges, and regulatory and safety challenges. PE-owned hospitals were more likely than other for-profit hospitals to report staff-training challenges.

**Figure 2.**
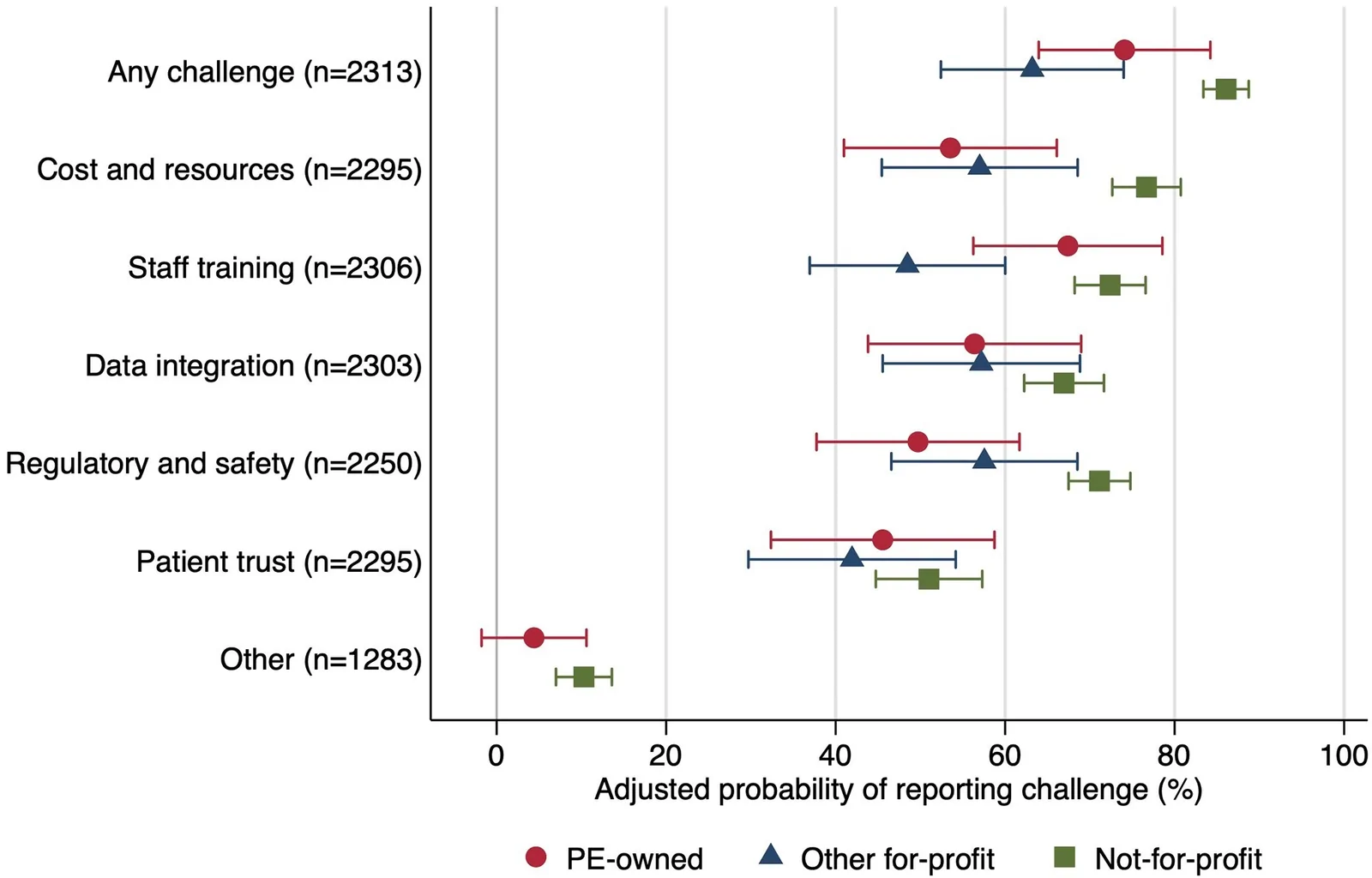
Exploratory adjusted probability of reporting artificial intelligence implementation challenges by hospital ownership. Probabilities derive from separate mixed-effects logistic regressions for reporting any challenge and for each of six challenge items, with state random intercepts and adjustment for hospital size, admissions, teaching status, accreditation, rural location, and market concentration. Each model includes hospitals answering the corresponding item. Error bars show 95% confidence intervals. The other for-profit estimate for “Other” is not estimable because none of 69 respondents reported it. PE indicates private equity.

**Table 3.** Exploratory response to and reporting of artificial intelligence implementation challenges by hospital ownership.

Challenge measure	PE-owned, % (95% CI)	Other FP, % (95% CI)	NFP, % (95% CI)	PE vs other FP, pp (95% CI); *P* value	PE vs NFP, pp (95% CI); *P* value	NFP vs other FP, pp (95% CI); *P* value
Challenge-grid response, all linked hospitals, adjusted % (*n* = 4305)	35.3 (15.6 to 55.0)	22.9 (−1.3 to 47.2)	58.5 (52.1 to 65.0)	12.4 (2.4 to 22.4); 0.015	−23.2 (−39.4 to −7.1); 0.005	35.6 (14.5 to 56.6); <0.001
*Among item respondents*						
Any challenge (*n* = 2313)	74.1 (64.0 to 84.2)	63.2 (52.4 to 74.0)	86.1 (83.4 to 88.7)	10.9 (−1.8 to 23.6); 0.093	−12.0 (−21.6 to −2.4); 0.014	22.9 (12.8 to 32.9); <0.001
Cost and resources (*n* = 2295)	53.5 (41.0 to 66.1)	57.0 (45.4 to 68.6)	76.7 (72.6 to 80.7)	−3.5 (−18.1 to 11.1); 0.642	−23.1 (−34.8 to −11.5); <0.001	19.7 (9.2 to 30.2); <0.001
Staff training (*n* = 2306)	67.4 (56.2 to 78.6)	48.5 (36.9 to 60.0)	72.4 (68.2 to 76.6)	18.9 (5.0 to 32.8); 0.008	−5.0 (−15.4 to 5.5); 0.349	23.9 (13.3 to 34.5); <0.001
Data integration (*n* = 2303)	56.4 (43.8 to 69.0)	57.2 (45.5 to 68.8)	67.0 (62.2 to 71.7)	−0.8 (−15.7 to 14.1); 0.917	−10.6 (−22.3 to 1.2); 0.079	9.8 (−0.9 to 20.5); 0.074
Regulatory and safety (*n* = 2250)	49.7 (37.7 to 61.7)	57.6 (46.6 to 68.5)	71.1 (67.5 to 74.8)	−7.8 (−22.3 to 6.6); 0.288	−21.4 (−32.9 to −9.9); <0.001	13.6 (3.2 to 24.0); 0.010
Patient trust (*n* = 2295)	45.6 (32.4 to 58.7)	41.9 (29.7 to 54.2)	51.0 (44.7 to 57.3)	3.6 (−11.5 to 18.8); 0.640	−5.5 (−17.4 to 6.5); 0.372	9.1 (−1.8 to 20.0); 0.103
Other (*n* = 1214)	4.4 (−1.8 to 10.6)	NE	10.3 (7.0 to 13.6)	NE	−5.9 (−12.4 to 0.6); 0.076	NE

The challenge-grid-response model included all 4305 linked hospitals and was estimated using logistic regression with state-clustered standard errors. Challenge-specific models included hospitals with a nonmissing response to the relevant item and were estimated using mixed-effects logistic regression with a state random intercept. All models adjusted for hospital bed size, admissions, rural location, teaching status, accreditation, and market concentration. Estimates are adjusted probabilities and percentage-point differences with 95% confidence intervals. Unadjusted *P* values are shown; statistical-significance statements use Bonferroni-adjusted *P* values for the three ownership contrasts within each outcome. Ownership was classified using the Kim et al. deal record definition.

AI, artificial intelligence; CI, confidence interval; FP, for-profit; NFP, not-for-profit, including government hospitals; PE, private equity; pp, percentage points.

Sensitivity analyses generally supported higher any and clinical AI adoption among PE-owned than other for-profit hospitals (see Supplementary Materials 2-6). The strict stage-5-vs-stage-1 comparison was directionally similar but not statistically significant. Propensity score matching produced average treatment effects on the treated of 23.4 percentage points for any AI and 25.1 percentage points for clinical AI in the 3:1, 0.10-caliper specification; operational AI remained nonsignificant. Coding nonresponse as non-adoption lowered absolute probabilities and resulted in lower estimated adoption among PE-owned than not-for-profit hospitals, reflecting the large ownership difference in module response. With region fixed effects and health-system-clustered standard errors, PE-vs-other-for-profit differences were 19.6 percentage points for any AI (95% CI, −2.8 to 42.1; *P* = 0.087) and 22.3 percentage points for clinical AI (95% CI, −0.5 to 45.1; *P* = 0.055). Moreover, excluding formerly PE-owned hospitals from the for-profit comparison group left the differences positive but no longer significant (9.4 percentage points for any AI, *P* = 0.281; 14.1 percentage points for clinical AI, *P* = 0.176), and the operational model did not converge.

## Discussion

PE firms have expanded their involvement in the U.S. health care delivery system, with researchers, regulators, and policymakers highlighting concerns arising from PE's emphasis on profitability.^37^ We provide the first evaluation of the association between PE hospital ownership and clinical and operational AI adoption. Using the Kim et al. deal-record database, PE-owned and not-for-profit hospitals had similar adjusted adoption of any and clinical AI, and both exceeded other for-profit hospitals. Operational adoption did not differ significantly by ownership. Results were consistent across most sensitivity analyses, although confidence intervals included the null after health-system clustering.

Our study is consistent with previous research on hospital ownership and technological investments. For-profit hospitals may respond rapidly when technologies offer clear financial returns,^32^ whereas not-for-profit hospitals may invest more in technologies that support clinical quality or organizational missions.^29-31^ At the same time, not-for-profit hospitals, many of which are large academic medical centers in the United States, have expanded their investment portfolios in the last decade, holding nearly $300 billion in investments in 2023.^38^ Recent evidence suggests that hospitals may be strategically investing in AI technology that can increase billing and coding intensity to increase revenues.^22^ While beyond the scope of this study, future research must examine whether investment gains experienced by not-for-profit hospitals help fund investment in AI technologies, and the downstream implications on clinical care patterns and patient outcomes.

Proponents of PE investments often assert that PE brings operational modernization capabilities and capital access unavailable to standalone facilities.^14-16^ However, PE firms’ use of debt financing has been associated with hospital closures and bankruptcies,^39^ which may constrain discretionary investment. The higher adoption of any and clinical AI among PE hospitals than among other for-profit hospitals is consistent with, but does not establish, a PE-related investment advantage. One explanation consistent with our findings is that the returns to clinical applications are more immediate than those to operational ones. Prior evidence that PE-acquired hospitals selectively added robotic surgery and digital mammography suggests that revenue-generating clinical technology, rather than revenue-cycle automation, is where acquisition capital is directed.^33^ It is possible that observed patterns may also reflect pre-existing infrastructure, organizational strategy, or correlated decisions within health systems.

Not-for-profit respondents reported more challenges overall and were more likely than PE-owned hospitals to identify cost and resource and regulatory and safety barriers. PE-owned hospitals were more likely than other for-profit hospitals to identify staff-training barriers. These patterns may reflect differences in the breadth or maturity of implementation and the opportunity to recognize barriers rather than weaker implementation capacity. Differential challenge response precludes interpreting fewer reported barriers as evidence of a capital-access advantage.

Several policy mechanisms can align investor incentives and technology adoption that serves patient care. First, regulators could tie reimbursement more directly to technology-enabled quality improvements, making AI investments financially attractive regardless of ownership structure. Second, public reporting of technology adoption alongside patient experience and safety metrics can support transparency and enable market-based accountability.^40^ Third, regulators could mandate minimum technology investment commitments that go beyond AI applications as conditions for acquisition approval.^41^ Taken together, our findings offer nuanced evidence to inform the ongoing policy debate on PE investments in hospitals.^39^

This study has several limitations. First, its cross-sectional design cannot establish whether PE ownership caused adoption; observed differences may reflect selection, pre-acquisition infrastructure, or organizational strategy. Because data on AI adoption were not available before 2024, we are unable to conduct a longitudinal analysis of whether PE ownership causes greater AI adoption over time. Second, AI use was self-reported and response rates were low and ownership dependent: 31.3% of PE-owned, 25.2% of other for-profit, and 57.7% of not-for-profit hospitals responded. Challenge response was also selective, and item-specific challenge comparisons remain vulnerable to differences between respondents and nonrespondents. Third, full integration is a binary endpoint that does not capture implementation stage, quality, workflow use, patient outcomes, or vendor-integrated tools. Fourth, the PE respondents represented 23 health-system clusters. Confidence intervals included the null after health-system clustering, and within-system dependence therefore remains a material source of uncertainty.

## Conclusion

Among United States general acute care hospitals responding to the 2024 AHA AI questions, PE-owned hospitals had higher adjusted adoption of any and clinical AI than other for-profit hospitals and similar adoption to not-for-profit hospitals. Operational adoption did not differ significantly by ownership. As PE firms expand their involvement in the U.S. health care system, our findings provide a preliminary empirical basis for evaluating the claim that PE investments drive technological improvements in health care.

## Supplementary Material

qxag261_Supplementary_Data

## Data sharing statement

Information on private equity ownership for United States hospitals is publicly available here: https://github.com/sungilkim94/Kim-PE-Data/blob/main/Kim-et-al-Private-Equity-Deal-List-with-Exit.dta. The American Hospital Association data that support this study are available under license and are not publicly available.
