# Supplementary material for: Private equity ownership and artificial intelligence adoption by hospitals in the United States
Source: Health Aff Sch. 2026 Sep 25;4(10):qxag261. doi: 10.1093/haschl/qxag261 (PMC13648208; doi:10.1093/haschl/qxag261)
Supplement: Supplementary file 1 [file qxag261_supplementary_data.zip › Supplementary Material 1.docx]

**Supplementary Material 1**. Mapping of study variables to 2024 AHA Annual Survey questions.

Data source: American Hospital Association Annual Survey 2024 Survey Instrument available at: <https://www.ahadata.com/system/files/media/file/2025/11/FY2024AnnualSurveyInstrument.pdf>

Primary outcome variable

**Artificial Intelligence (AI) adoption** – survey section G. Information Technology and Cybersecurity, Question 2 (Artificial Intelligence). Question 2a: Clinical AI implementation “How would you describe your current level of AI implementation in the following clinical areas?”. Response options include (1) not implementing, (2) exploring, (3) piloting/testing, (4) expanding, (5) fully integrated, (0) don’t know. Clinical domains include AI-assisted diagnostics (including imaging & early detection; predictive analytics for patient care (including outcomes & deterioration); clinical decision support tools; AI-assisted surgery; AI-powered patient communication and education; AI-driven population health management; predictive models for resource allocation during emergencies; other, please specify. Question 2b: Operational AI implementation "How would you describe your current level of AI implementation in the following operational areas?". Operational AI domains include revenue cycle management (e.g., billing, claims processing); supply chain optimization; staff scheduling and workforce management; patient flow and demand forecasting; optimizing operational efficiency; other, please specify.

Variable definition for analysis:

- Any AI adoption: Binary variable coded as 1 if hospital reported "Fully Integrated" (option 5) for at least one clinical OR operational AI domain; 0 otherwise
- Clinical AI adoption: Binary variable coded as 1 if "Fully Integrated" for at least one clinical AI domain
- Operational AI adoption: Binary variable coded as 1 if "Fully Integrated" for at least one operational AI domain

Secondary outcome variable

**Challenges in AI implementation** – survey section G. Information Technology and Cybersecurity, Question 2c. "Has your hospital encountered the following challenges in implementing AI in clinical or operational areas?". Response options: (1) Yes, (2) No. Challenge categories: Cost and resource requirements, staff training and upskilling, data integration and interoperability, regulatory compliance and safety concerns, Patient trust and acceptance, other, please specify.

Variable definition for analysis:

- Any challenge: Binary variable coded as 1 if hospital reported "Yes" to at least one challenge category
- Specific challenges: Binary variables for each challenge type (1=Yes, 0=No) among hospitals reporting any challenge

Hospital characteristics variables

**Organizational control (ownership type)** - survey section: B. Organizational Structure, Question 1 (Control). "Indicate the type of organization that is responsible for establishing policy for overall operation of your hospital. CHECK ONLY ONE:”. Categories used in analysis: For-profit - Codes 31 (Individual), 32 (Partnership), or 33 (Corporation); Non-profit - Codes 12 (State), 13 (County), 14 (City), 15 (City-County), 16 (Hospital district or authority), 21 (Church-operated), 23 (Other not-for-profit including NFP Corporation), 40 (Department of Defence), 44 (Public Health Service), 45 (Veterans Affairs), 46 (Federal other), 47 (PHS Indian Service), 48 (Department of Justice)

**Hospital size** - survey section: E. Total Facility Beds and Utilization, Question 2b. "Beds set up and staffed for use at the end of the reporting period".

Variable definition for analysis:

- Small: <100 beds
- Medium: 100–300 beds
- Large: >300 beds (reference category)

**Hospital volume** – survey section: E. Total Facility Beds and Utilization, Question 2e. "Admissions (exclude newborns; include neonatal & swing admissions)".

Variable definition for analysis:

- Total number of admissions during the reporting period (continuous variable)

**Teaching status** – survey section: Multiple sources used to identify teaching hospitals.

Variable definition for analysis:

- Hospital classified as teaching if it declared membership in the Association of American Medical Colleges (AAMC), or medical school affiliation reported to American Medical Association (AMA)
- Binary variable: 1=Teaching hospital, 0=non-teaching hospital

**Accreditation status** – survey section: Hospital accreditation by Joint Commission

Binary variable: 1=Accredited by Joint Commission, 0=Not accredited

**Rural status** – Core-Based Statistical Area classification. "CBSATYPE"; 1) Metropolitan (urban), 2) Micropolitan (small city/town), 3) Rural

Variable definition for analysis:

- Binary variable: 1 = Rural, 0 otherwise.
