# Supplementary material for: Private equity ownership and artificial intelligence adoption by hospitals in the United States
Source: Health Aff Sch. 2026 Sep 25;4(10):qxag261. doi: 10.1093/haschl/qxag261 (PMC13648208; doi:10.1093/haschl/qxag261)
Supplement: Supplementary file 1 [file qxag261_supplementary_data.zip › Supplementary Material 2.docx]

**Supplementary Material 2. Alternative nonresponse coding.**

| **Coding / specification** | **Outcome** | **N** | **PE vs other FP, pp (95% CI); P value** | **PE vs NFP, pp (95% CI); P value** | **NFP vs other FP, pp (95% CI); P value** |
| --- | --- | --- | --- | --- | --- |
| Primary respondent-only | Any AI | 2,290 | 24.51 (11.19 to 37.84); <0.001 | 0.95 (-10.92 to 12.82); 0.875 | 23.57 (16.26 to 30.87); <0.001 |
| Primary respondent-only | Clinical AI | 2,275 | 27.53 (13.99 to 41.07); <0.001 | 3.54 (-8.59 to 15.67); 0.568 | 23.99 (17.00 to 30.99); <0.001 |
| Primary respondent-only | Operational AI | 2,205 | -0.13 (-4.71 to 4.44); 0.955 | -3.77 (-7.70 to 0.16); 0.060 | 3.64 (-0.13 to 7.40); 0.058 |
| Wholly blank AI module coded 0 | Any AI | 4,197 | 6.35 (2.70 to 10.00); <0.001 | -8.62 (-12.48 to -4.76); <0.001 | 14.97 (11.16 to 18.79); <0.001 |
| Wholly blank AI module coded 0 | Clinical AI | 4,182 | 6.94 (3.12 to 10.77); <0.001 | -7.52 (-11.38 to -3.66); <0.001 | 14.47 (10.79 to 18.14); <0.001 |
| Wholly blank AI module coded 0 | Operational AI | 4,112 | 0.49 (-0.41 to 1.39); 0.288 | -2.73 (-4.42 to -1.05); 0.001 | 3.22 (1.47 to 4.98); <0.001 |
| All missing outcomes coded 0 | Any AI | 4,305 | 5.85 (2.45 to 9.26); <0.001 | -8.65 (-12.34 to -4.97); <0.001 | 14.51 (10.82 to 18.19); <0.001 |
| All missing outcomes coded 0 | Clinical AI | 4,305 | 6.32 (2.80 to 9.83); <0.001 | -7.66 (-11.30 to -4.02); <0.001 | 13.98 (10.45 to 17.50); <0.001 |
| All missing outcomes coded 0 | Operational AI | 4,305 | 0.40 (-0.42 to 1.21); 0.339 | -2.66 (-4.26 to -1.05); 0.001 | 3.05 (1.38 to 4.72); <0.001 |

Note: All specifications used mixed-effects logistic regression with a state random intercept and adjustment for hospital size, admissions, rural location, teaching status, accreditation, and market concentration. In the primary respondent-only analysis, an outcome was defined only when a hospital provided at least one substantive stage 1–5 response in the relevant AI domain. Under “wholly blank AI module coded 0,” hospitals providing neither a substantive response nor a “don’t know” response across all 12 AI items were classified as non-adopters; “don’t know”-only modules and domain-specific missing outcomes remained missing. Under “all missing outcomes coded 0,” every missing any-, clinical-, or operational-AI outcome was classified as non-adoption, including wholly blank modules, “don’t know”-only modules, and missing responses within a specific domain. Contrasts are expressed as percentage-point differences between the first- and second-listed groups. AI indicates artificial intelligence; CI, confidence interval; FP, for-profit; NFP, not-for-profit, including government hospitals; PE, private equity; pp, percentage points.
