# Supplementary material for: Private equity ownership and artificial intelligence adoption by hospitals in the United States
Source: Health Aff Sch. 2026 Sep 25;4(10):qxag261. doi: 10.1093/haschl/qxag261 (PMC13648208; doi:10.1093/haschl/qxag261)
Supplement: Supplementary file 1 [file qxag261_supplementary_data.zip › Supplementary Material 3.docx]

**Supplementary Material 3. Alternative outcome definitions and inverse-response weighting.**

| **Definition / specification** | **Outcome** | **N** | **PE vs other FP, pp (95% CI); P value** | **PE vs NFP, pp (95% CI); P value** | **NFP vs other FP, pp (95% CI); P value** |
| --- | --- | --- | --- | --- | --- |
| Stage 5 vs stage 1 | Any AI | 993 | 12.38 (-6.94 to 31.69); 0.209 | -16.30 (-28.96 to -3.64); 0.012 | 28.68 (13.56 to 43.80); <0.001 |
| No “don’t know” responses | Any AI | 1,860 | 25.25 (10.63 to 39.86); <0.001 | 2.94 (-10.16 to 16.04); 0.660 | 22.31 (14.54 to 30.07); <0.001 |
| Ordinal stages 1–5: Pr (stage 5) | Any AI | 2,290 | 12.85 (4.52 to 21.19); 0.003 | -8.33 (-17.00 to 0.35); 0.060 | 21.18 (15.18 to 27.19); <0.001 |
| Inverse-response weighting | Any AI | 2,285 | 15.87 (4.22 to 27.52); 0.008 | -7.92 (-19.43 to 3.60); 0.178 | 23.79 (18.16 to 29.41); <0.001 |

Note: The strict binary outcome compares hospitals whose highest substantive response was stage 5 with those whose highest response was stage 1, excluding stages 2–4. The complete-response specification retains the primary any-AI outcome but excludes hospitals reporting “don’t know” for any AI item. Estimates are covariate-adjusted probability differences between the first- and second-listed groups; reported P values are not adjusted for multiple comparisons. The strict and complete-response specifications used mixed-effects logistic regression with a state random intercept. The ordinal specification used ordered logistic regression with state-clustered standard errors and reports the adjusted probability of stage 5. The inverse-response-weighted specification used weighted logistic regression with region fixed effects and state-clustered standard errors; weights equal the inverse predicted probability of providing a substantive AI-module response, estimated from ownership and observed hospital and market characteristics. AI indicates artificial intelligence; CI, confidence interval; FP, for-profit; NFP, not-for-profit, including government hospitals; PE, private equity; pp, percentage points.
