# Supplementary material for: Private equity ownership and artificial intelligence adoption by hospitals in the United States
Source: Health Aff Sch. 2026 Sep 25;4(10):qxag261. doi: 10.1093/haschl/qxag261 (PMC13648208; doi:10.1093/haschl/qxag261)
Supplement: Supplementary file 1 [file qxag261_supplementary_data.zip › Supplementary Material 4.docx]

**Supplementary Material 4. Region fixed effects, reduced adjustment, Firth estimation, and exclusion of formerly private equity owned hospitals within the for-profit sector.**

| **Outcome** | **Specification** | **N** | **States, No.** | **PE-owned, % (95% CI)** | **Other FP, % (95% CI)** | **PE vs other FP, pp (95% CI)** | **P value** | **Estimation diagnostic** |
| --- | --- | --- | --- | --- | --- | --- | --- | --- |
| Any AI | Full covariates + region FE | 159 | 19 | 38.9 (25.8 to 52.0) | 8.3 (5.1 to 11.6) | 30.5 (17.2 to 43.9) | <0.001 | 16 observations outside fitted sample |
| Any AI | Reduced covariates + region FE | 159 | 19 | 34.6 (22.3 to 46.9) | 9.4 (6.4 to 12.5) | 25.2 (12.6 to 37.8) | <0.001 | 16 observations outside fitted sample |
| Any AI | Firth, reduced covariates | 175 | — | 31.4 (20.9 to 41.9) | 9.8 (4.1 to 15.6) | 21.5 (9.5 to 33.6) | <0.001 | Full outcome-specific sample |
| Any AI | Mixed effects; former PE controls excluded | 150 | 26 | 12.8 (−11.5 to 37.1) | 3.4 (−4.9 to 11.8) | 9.4 (−7.7 to 26.5) | 0.281 | 25 former PE controls excluded; full eligible sample fitted |
| Clinical AI | Full covariates + region FE | 156 | 19 | 39.3 (25.7 to 52.9) | 6.6 (4.0 to 9.2) | 32.7 (18.6 to 46.8) | <0.001 | 15 observations outside fitted sample |
| Clinical AI | Reduced covariates + region FE | 156 | 19 | 35.5 (22.8 to 48.3) | 7.4 (4.6 to 10.1) | 28.2 (15.1 to 41.3) | <0.001 | 15 observations outside fitted sample |
| Clinical AI | Firth, reduced covariates | 171 | — | 32.2 (21.6 to 42.9) | 8.0 (2.7 to 13.4) | 24.2 (12.2 to 36.2) | <0.001 | Full outcome-specific sample |
| Clinical AI | Mixed effects; former PE controls excluded | 146 | 25 | 17.8 (−8.6 to 44.2) | 3.7 (−4.0 to 11.4) | 14.1 (−6.3 to 34.5) | 0.176 | 25 former PE controls excluded; full eligible sample fitted |
| Operational AI | Full covariates + region FE | 101 | 16 | 14.9 (−7.5 to 37.3) | 8.6 (5.5 to 11.8) | 6.3 (−15.8 to 28.4) | 0.575 | 60 observations outside fitted sample |
| Operational AI | Reduced covariates + region FE | 101 | 16 | 11.4 (−9.0 to 31.7) | 10.5 (7.3 to 13.8) | 0.9 (−20.0 to 21.7) | 0.937 | 60 observations outside fitted sample |
| Operational AI | Firth, reduced covariates | 161 | — | 9.0 (2.2 to 15.9) | 7.9 (2.4 to 13.3) | 1.2 (−7.8 to 10.2) | 0.801 | Full outcome-specific sample |
| Operational AI | Mixed effects; former PE controls excluded | — | — | NE | NE | NE | NE | 25 former PE controls excluded; model did not converge |

Note: Adjusted probabilities were standardized over the observed covariate distribution in each estimation sample. Full adjustment included hospital size, admissions, rural location, teaching status, accreditation, and market concentration; reduced adjustment included hospital size, rural location, and teaching status. Conventional logistic models included region fixed effects and state-clustered standard errors. Firth penalized-likelihood models used the reduced covariate set without region fixed effects or clustering. In the comparison-group sensitivity analysis, hospitals appearing in the Kim et al deal file but no longer actively PE-owned in 2024 were excluded from the other for-profit group; current PE ownership continued to follow the Kim et al deal-record definition. This sensitivity analysis used mixed-effects logistic regression with the full covariate set and a state random intercept, with adjusted probabilities calculated using the fixed component of the model. The operational-AI exclusion model did not converge and was not reported. Confidence intervals are unadjusted delta-method Wald intervals and were not truncated at 0% or 100%. P values are two-sided and unadjusted for multiple comparisons. AI indicates artificial intelligence; CI, confidence interval; FE, fixed effects; FP, for-profit; NE, not estimable; PE, private equity; and pp, percentage points.
