# Supplementary material for: Private equity ownership and artificial intelligence adoption by hospitals in the United States
Source: Health Aff Sch. 2026 Sep 25;4(10):qxag261. doi: 10.1093/haschl/qxag261 (PMC13648208; doi:10.1093/haschl/qxag261)
Supplement: Supplementary file 1 [file qxag261_supplementary_data.zip › Supplementary Material 5.docx]

**Supplementary Material 5. Health-system-clustered inference.**

| **Outcome** | **N** | **System clusters** | **PE vs other FP, pp (95% CI); P value** | **PE vs NFP, pp (95% CI); P value** | **NFP vs other FP, pp (95% CI); P value** |
| --- | --- | --- | --- | --- | --- |
| Any AI | 2,285 | 836 | 19.62 (-2.82 to 42.06); 0.087 | -7.09 (-28.35 to 14.18); 0.514 | 26.71 (17.12 to 36.29); <0.001 |
| Clinical AI | 2,270 | 827 | 22.30 (-0.47 to 45.07); 0.055 | -4.50 (-26.32 to 17.31); 0.686 | 26.81 (18.01 to 35.60); <0.001 |
| Operational AI | 2,200 | 816 | 0.88 (-8.45 to 10.22); 0.853 | -8.35 (-17.43 to 0.73); 0.072 | 9.23 (2.13 to 16.34); 0.011 |

Note: Models use full covariates, region fixed effects, and standard errors clustered by health-system name. Hospitals without a system name form individual clusters. Five territory observations were omitted because of perfect prediction. AI is artificial intelligence; CI, confidence interval; FE, fixed effects; FP, for-profit; NFP, not-for-profit, including government hospitals; PE, private equity; pp, percentage points; SE, standard error.
