# Supplementary material for: Private equity ownership and artificial intelligence adoption by hospitals in the United States
Source: Health Aff Sch. 2026 Sep 25;4(10):qxag261. doi: 10.1093/haschl/qxag261 (PMC13648208; doi:10.1093/haschl/qxag261)
Supplement: Supplementary file 1 [file qxag261_supplementary_data.zip › Supplementary Material 6.docx]

**Supplementary Material 6. Propensity-score matching.**

| **Outcome** | **Ratio; caliper** | **N analysis; matched PE** | **Matched adoption, % PE / other FP** | **ATT, pp (95% CI)** | **P value** | **Mean absolute SMD, % before to after** | **Maximum post-match absolute SMD, %; balanced n/9** |
| --- | --- | --- | --- | --- | --- | --- | --- |
| Any AI | 3:1; 0.10 | 175; 67 | 31.3 / 8.0 | 23.38 (9.36 to 37.40) | 0.001 | 18.7 to 6.3 | 20.9; 8/9 |
| Any AI | 1:1; 0.25 | 175; 67 | 31.3 / 13.4 | 17.91 (3.61 to 32.22) | 0.014 | 18.7 to 14.6 | 42.4; 4/9 |
| Any AI | 3:1; 0.25 | 175; 67 | 31.3 / 8.0 | 23.38 (9.36 to 37.40) | 0.001 | 18.7 to 6.3 | 20.9; 8/9 |
| Any AI | 5:1; 0.25 | 175; 67 | 31.3 / 7.8 | 23.58 (10.19 to 36.97) | <0.001 | 18.7 to 5.4 | 13.5; 8/9 |
| Clinical AI | 3:1; 0.10 | 171; 65 | 32.3 / 7.2 | 25.13 (11.54 to 38.71) | <0.001 | 18.8 to 5.5 | 11.6; 8/9 |
| Clinical AI | 1:1; 0.25 | 171; 65 | 32.3 / 7.7 | 24.62 (10.19 to 39.04) | <0.001 | 18.8 to 7.5 | 16.2; 5/9 |
| Clinical AI | 3:1; 0.25 | 171; 65 | 32.3 / 7.2 | 25.13 (11.54 to 38.71) | <0.001 | 18.8 to 5.5 | 11.6; 8/9 |
| Clinical AI | 5:1; 0.25 | 171; 65 | 32.3 / 7.6 | 24.72 (10.88 to 38.56) | <0.001 | 18.8 to 4.7 | 9.3; 9/9 |
| Operational AI | 3:1; 0.10 | 161; 63 | 7.9 / 5.8 | 2.12 (-8.34 to 12.58) | 0.692 | 18.9 to 7.7 | 18.4; 7/9 |
| Operational AI | 1:1; 0.25 | 161; 63 | 7.9 / 1.6 | 6.35 (-2.63 to 15.33) | 0.166 | 18.9 to 9.3 | 17.2; 4/9 |
| Operational AI | 3:1; 0.25 | 161; 63 | 7.9 / 5.8 | 2.12 (-8.34 to 12.58) | 0.692 | 18.9 to 7.7 | 18.4; 7/9 |
| Operational AI | 5:1; 0.25 | 161; 63 | 7.9 / 5.7 | 2.22 (-7.24 to 11.69) | 0.645 | 18.9 to 5.8 | 13.7; 7/9 |

Note: Matching compares PE-owned with other for-profit hospitals using nearest-neighbor matching with replacement, ties, and common support. Propensity scores include hospital size, rural location, teaching status, accreditation, county HHI, and region indicators. ATT denotes the average treatment effect on the treated. AI is artificial intelligence; ATT, average treatment effect on the treated; CI, confidence interval; FP, for-profit; HHI, Herfindahl-Hirschman Index; PE, private equity; pp, percentage points; SMD, standardized mean difference.
